# Serum iron level is independently associated with sarcopenia: a retrospective study

**DOI:** 10.1038/s41598-024-61429-0

**Published:** 2024-05-08

**Authors:** Meiying Huang, Bingqing Xu, Yihui Xu, Kaiyu Zhang, Wenyu Zhu, Xiaoyi Lian, Zhe Chen, Minhong Wang, Lei Liu, Zhengli Guo

**Affiliations:** 1 Department of Gerontology, Affiliated Kunshan Hospital of Jiangsu University, Kunshan, 215300 Jiangsu China; 2 Chinese Academy of Medical Science and Peking Union Medical College, Beijing, China; 3 Laboratory of Cough, Affiliated Kunshan Hospital of Jiangsu University, Kunshan, Jiangsu China; 4 Department of Gerontology, Suzhou Municipal Hospital, Suzhou, 215300 Jiangsu China

**Keywords:** Geriatrics, Risk factors

## Abstract

Sarcopenia greatly reduces the quality of life of the elderly, and iron metabolism plays an important role in muscle loss. This study aimed to investigate the association between iron status and sarcopenia. A total of 286 adult patients hospitalized between 2019 and 2021 were included in this study, of which 117 were diagnosed with sarcopenia. Serum iron, total iron binding capacity (TIBC), transferrin, and transferrin saturation levels were compared between groups with and without sarcopenia and were included in the logistic analyses, with significant variables further included in the logistic regression model for the prediction of sarcopenia. Serum iron, TIBC, and transferrin levels decreased significantly in the sarcopenia group (p < 0.05), and were negatively associated with handgrip strength, relative skeletal muscle index, and multiple test performances (p < 0.05). Multivariate logistic analysis showed that sex, age, body mass index (BMI), and serum iron level were independent risk factors for sarcopenia. In the final logistic regression model, male sex (odds ratio [OR] 3.65, 95% confidence interval [CI] 1.67–7.98), age > 65 years (OR 5.40, 95% CI 2.25–12.95), BMI < 24 kg/m^2^ (OR 0.17, 95% CI 0.08–0.36), and serum iron < 10.95 μmol/L (OR 0.39, 95% CI 0.16–0.93) were included. Our study supported the impact of iron metabolism on muscle strength and performance.

## Introduction

Sarcopenia is characterized by age-related decline in skeletal muscle mass, strength, and physical performance^1,2^. As an underlying risk factor for falls, frailty, disability, and death, sarcopenia is a serious health hazard in the older population^3,4^. Its prevalence varies between 10 and 27%, as reported in different studies, and increases significantly with age^5^. Many studies have investigated the epidemiological features, diagnosis, and clinical impact of sarcopenia in various populations^6–10^. However, its pathogenesis remains to be elucidated, and the identification of serum markers could provide important clues and assist in its diagnosis and prevention.

Recently, the association between the dysregulation of iron metabolism and muscle loss has attracted much attention. Iron deficiency is associated with skeletal muscle abnormalities and dysfunction in patients with heart failure^11,12^. On the other hand, iron overload has also been suggested as a risk factor for muscle loss. Studies have reported that an increased prevalence of sarcopenia is associated with higher serum ferritin levels in older women in Korea and in adults in the United States^13,14^. Similar results were also reported in studies on hemodialysis patients and patients with gastric cancer, where higher ferritin levels correlated with loss of muscle strength and quality^15,16^. Similarly, in animal and cellular studies, both iron overload and iron deficiency have been suggested as causes of poor muscle mass and function^17–21^.

In the above clinical studies, serum ferritin was used as the main indicator of systemic iron load and was inconsistently associated with sarcopenia^11–13,15,16^. In clinical practice, iron status is indicated by several markers, including serum ferritin, transferrin, total iron binding capacity (TIBC), and transferrin saturation (TSAT) levels, which are much less frequently included in sarcopenia studies. Therefore, we aimed to further investigate the relationship between iron status and sarcopenia, and whether iron status measurements can be used in the prediction of sarcopenia.

## Methods

### Patient population

A total of 343 patients aged 50 years and above, hospitalized at the Affiliated Kunshan Hospital of Jiangsu University between October 2019 and August 2021, were included in this study. Exclusion criteria were (1) severe heart failure, liver failure, or kidney failure; (2) acute coronary syndrome or stroke; (3) therapy for anemia or transfusion within 12 months preceding hospitalization; (4) muscular or neuromuscular disorders; and (5) dementia or cognitive impairment. After exclusion, a total of 286 patients were included in the final analysis.

Sample size estimation was conducted based on statistical power analysis using G-power software (G Power 3.1.9.7). We performed sample size estimation for logistic regression analysis under the following assumptions, based on previous studies: a significance level of 0.05, statistical power of 0.80, and an odds ratio of 2.5^11,13,14^. A sample size of 192 was needed to ensure statistically significant results. After exclusion, the final sample size for the study was determined to be 286 patients, which is deemed adequate to fulfill the objectives of our study and provides sufficient statistical power to detect the expected effects.

The study protocol was approved by the local ethics committee (Jiangsu University, IEC-C-011-A04), and all participants provided written informed consent. This study was conducted in accordance with the principles of the Declaration of Helsinki.

### Data collection

Clinical data were collected between October 2019 and August 2021 by the hospital staff, and retrospectively analyzed. All baseline characteristics, except for the length of hospitalization, were obtained on the first day of hospitalization. Body mass index (BMI) and fat percentage were measured using dual-energy X-ray absorptiometry (DXA) scans performed using Hologic Discovery DXA and software version 13.5.3. Hypertension was defined as systolic blood pressure (SBP) ≥ 140 mmHg or diastolic blood pressure (DBP) ≥ 90 mmHg or the need for antihypertensive medications to maintain normal blood pressure. Diabetes mellitus was diagnosed according to the criteria proposed by the World Health Organization in 1999 and 2011 or established by previous diagnoses^22,23^.

Blood tests were performed with fresh venous blood samples taken after overnight (> 8 h) fasting within 24 h of hospitalization. For iron status measurements, serum ferritin (μg/L), serum iron (μmol/L), transferrin (g/L), and TIBC (μmol/L) were directly measured, while TSAT was calculated as (serum iron/TIBC) × 100% and expressed as a percentage. Serum iron and TIBC levels were measured based on a colorimetric method using a Cobas c system (Roche). Transferrin levels were measured using a turbidimetric inhibitory immunoassay method with the Cobas c system (Roche). Serum ferritin levels were measured using a chemiluminescence method with the Elecsys 2010 system (Roche).

Sarcopenia was diagnosed according to the Asian Working Group for Sarcopenia criteria proposed in 2019^1^. The relative skeletal muscle index (RSMI) was measured using DXA scans, and handgrip strength (dominant hand) was measured using a CAMRY EH101 electronic hand dynamometer. In the 6-m walk test, patients with a usual gait speed < 1.0 m/s were classified as having positive results. In 5-time chair stand test, patients were asked to rise from a chair (standard height: 43 cm) five times with arms folded across their chests, and results with a total time of ≥ 12 s were defined as positive. In the balance test, patients were asked to stand first with feet aligned side-by-side, then with one heel at the midpoint of the other foot, and finally with feet aligned in a line, heel to toe. Failure to maintain balance in any of the three postures for > 10 s was defined as a positive result.

### Statistical analysis

Statistical analyses were performed using R 4.1.2 (https://www.r-project.org/) software. Descriptive characteristics are presented as means (standard deviation [SD]), median (interquartile range [IQR]), or percentages. Student’s t-test, χ square test, and Mann–Whitney U-test were performed to analyze intergroup differences, and Spearman correlation analysis was used to measure the correlation between variables. Logistic analysis was used to examine the association between the iron status indices and sarcopenia. Baseline characteristics, hemoglobin and albumin levels, and iron status measurements were included in the univariate analysis for the selection of significant (p < 0.05) independent variables for multivariate analysis. The continuous variables included in the logistic regression analysis were transformed into binary variables using cutoff values determined either by clinical significance or by receiver operating characteristic (ROC) curves. Although significant in the univariate analysis, TSAT was not included in the multivariate analysis due to interaction concerns, given that it was calculated as serum iron/TIBC. To develop a logistic regression model for the prediction of sarcopenia, patients were randomly assigned in a 6:4 ratio to a training cohort (n = 175) and a validation cohort (n = 111). The logistic regression model was developed with the training cohort data, using the four variables (sex, age, BMI, and serum iron) confirmed to be significant (P < 0.05) in the multivariate analysis, and tested in the validation cohort.

### Ethics approval and consent to participate

The study protocol was approved by the local ethics committee (Jiangsu University), and all participants provided written informed consent. This study was conducted in accordance with the principles of the Declaration of Helsinki.

## Results

### Patient characteristics

A total of 286 hospitalized patients (mean age = 70.6 years, male/female ratio, 107/179) were included in the analysis, of which 117 (40.9%) were diagnosed with sarcopenia (Table 1). In total, 122 (48.7%), 148 (51.7%), and 21 (7.3%) patients showed positive results in the 6-m walk test, 5-time chair stand test, and balance test, respectively, and patients with sarcopenia had more positive results in these tests than those without sarcopenia (p < 0.001 for the 6-m walk test and 5-time chair stand test, p = 0.003 for the balance test).

**Table 1:** Clinical characteristics of patients.

Characteristics	N = 286
Baseline characteristics	
Age (years)	70.6 ± 9.6
Male, n (%)	107 (37.4)
Length of hospitalization (days)	8 (6–11)
BMI (kg/m^2^)	23.4 ± 3.3
Fat percent	32.5% ± 7.0%
Hypertension, n (%)	158 (55.2)
Diabetes mellitus, n (%)	73 (25.5)
Cancer, n (%)	28 (9.7)
Blood test results	
Hemoglobin (g/L)	126.6 ± 15.4
RBC (*10^12^/L)	4.16 ± 0.49
WBC (*10^12^/L)	5.68 ± 1.82
Hematocrit (%)	38.7 (35.6–41.3)
Albumin (g/L)	39.7 ± 4.1
Iron status measurements	
Ferritin (μg/L)	204.5 (126.0–325.1)
Serum iron (μmol/L)	14.4 (11.0–18.1)
TIBC (μmol/L)	48.3 ± 8.7
TSAT (%)	29.2 (22.9–36.2)
Transferrin (g/L)	2.07 ± 0.38
Sarcopenia-related indices	
Sarcopenia, n (%)	117 (40.9)
SMI (kg)	14.43 ± 2.07
RSMI (kg/m^2^)	5.98 ± 1.06
Handgrip strength (kg)	21.1 (15.4–26.9)
6-m walk test, positive n (%)	122 (42.7)
5-time chair stand test, positive n (%)	148 (51.7)
Balance test, positive n (%)	21 (7.3)

Data are expressed as mean ± standard deviation for variables with normal distribution, and median (interquartile range) for variables with nonnormal distribution.

*BMI* body mass index, *RBC* red blood cells, *WBC* white blood cells, *TIBC* total iron binding capacity, *TSAT* transferrin saturation, *SMI* skeletal muscle index, *RSMI* relative skeletal muscle index.

### Comparison of clinical characteristics between groups with and without sarcopenia

When divided into sarcopenia and non-sarcopenia groups, patients showed significant differences in age, sex, BMI, and other clinical characteristics (Table 2). Patients with sarcopenia were older, more likely to be male, had a lower BMI, and had lower albumin and hemoglobin levels. Among serum measurements for iron metabolism, serum iron, TIBC, and transferrin levels were significantly lower in the sarcopenia group, while ferritin and TSAT levels showed no significant differences. The diabetes and hypertension rates did not differ between the two groups.

**Table 2:** Clinical characteristics between groups with or without sarcopenia.

	Non-sarcopenia (169)	Sarcopenia (117)	P
Age	67.0 ± 8.8	75.7 ± 8.2	< 0.001**
Male, n (%)	48 (28.4)	59 (50.4)	< 0.001**
BMI	24.8 ± 2.6	21.4 ± 3.1	< 0.001**
Diabetes	40 (23.7)	33 (28.2)	0.387
Hypertension	91 (53.8)	67 (57.3)	0.568
Albumin	40.6 ± 3.9	38.4 ± 4.1	< 0.001**
Hemoglobin	129.0 (121.0–138.3)	123.0 (110.9–135.0)	< 0.001**
Ferritin	201.9 (126.0–321.6)	211.0 (125.1–347.4)	0.627
Serum iron	14.7 (11.7–18.7)	13.0 (8.6–17.9)	0.007**
TIBC	50.2 ± 8.5	45.6 ± 8.3	< 0.001**
TSAT	29.7 (24.6–36.1)	28.1 (21.1–37.4)	0.207
Transferrin	2.15 ± 0.37	1.96 ± 0.38	< 0.001**

Student’s t-test was performed to analyze intergroup differences for variables with normal distribution, while χ square test was performed for sex, diabetes status, and hypertension, and Mann–Whitney U-test were performed for hemoglobin, ferritin, serum iron, and TSAT. Data are expressed as mean ± standard deviation for variables with normal distribution, and median (interquartile range) for variables with nonnormal distribution. (**P < 0.01).

*BMI* body mass index, *TIBC* total iron binding capacity, *TSAT* transferrin saturation.

### Relationship between iron status measurements and sarcopenia-related indices

Higher serum iron, TIBC, and transferrin levels were significantly associated with higher handgrip strength and RSMI and a lower risk of positive results in the 6-m walk test, 5-time chair stand test, and balance test (Table 3). TSAT levels were also significantly associated with handgrip strength, RSMI, and positive 5-time chair stand test results, while ferritin levels showed a significant correlation only with RSMI.

**Table 3:** Correlations between iron status measurements and sarcopenia-related indices.

	Handgrip strength	6-m walk test	5-time chair stand test	Balance test	RSMI
Ferritin	0.080	0.031	0.044	0.065	0.134*
Serum iron	0.229**	− 0.200**	− 0.181**	− 0.177**	0.228**
TIBC	0.123*	− 0.134*	− 0.145*	− 0.135*	0.131*
TSAT	0.206**	− 0.100	− 0.124*	− 0.088	0.190**
Transferrin	0.157**	− 0.171**	− 0.177**	− 0.141*	0.119*

This table shows Spearman r results, and significant correlations are indicated with asterisks (*P < 0.05; **P < 0.01).

*TIBC* total iron binding capacity, *TSAT* transferrin saturation, *RSMI* relative skeletal muscle index.

### Logistic regression analyses for sarcopenia as the dependent variable

Variables were included in logistic regression analyses as categorical variables, with cutoff values determined by clinical significance (age, BMI, albumin, and hemoglobin) or receiver operator characteristic curve analysis (ferritin, transferrin, serum iron, TIBC, and TSAT). In univariate logistic regression analysis, sex, age, BMI, albumin, hemoglobin, transferrin, serum iron, TIBC, and TSAT were significantly associated with the risk of sarcopenia. All significant variables, except TSAT (due to concerns of interaction), were included in the multivariate logistic regression analysis, and sex, age, BMI, and serum iron remained significant (Table 4).

**Table 4:** Univariate and multivariate analyses with sarcopenia as the dependent variable.

Variables	Univariate analysis		Multivariate analysis	
	OR (95% CI)	*P*	OR (95% CI)	*P*
Sex				
Female	Reference		Reference	
Male	2.564 (1.566–4.199)	< 0.001*	3.553 (1.872–6.745)	< 0.001*
Age (year)				
≤ 65	Reference		Reference	
> 65	5.297 (2.845–9.860)	< 0.001*	4.674 (2.219–9.844)	< 0.001*
BMI (kg/m^2^)				
≤ 24	Reference		Reference	
> 24	0.197 (0.116–0.333)	< 0.001*	0.168 (0.092–0.309)	< 0.001*
Hypertension				
No	Reference			
Yes	1.149 (0.714–1.847)	0.568		
Diabetes				
No	Reference			
Yes	1.267 (0.741–2.167)	0.387		
Albumin (g/L)				
≤ 40	Reference		Reference	
> 40	0.447 (0.275–0.725)	0.001*	1.277 (0.641–2.546)	0.487
Hemoglobin (g/L)				
≤ 120	Reference		Reference	
> 120	0.402 (0.241–0.671)	< 0.001*	0.591 (0.295–1.184)	0.138
Ferritin (ng/mL)				
≤ 217.6	Reference			
> 217.6	1.390 (0.965–2.234)	0.173		
Transferrin (g/L)				
≤ 1.86	Reference		Reference	
> 1.86	0.337 (0.198–0.571)	< 0.001*	0.748 (0.319–1.758)	0.481
Serum iron (μmol/L)				
≤ 10.95	Reference		Reference	
> 10.95	0.327 (0.187–0.571)	< 0.001*	0.482 (0.236–0.986)	0.041*
TIBC (μmol/L)				
≤ 47.05	Reference		Reference	
> 47.05	0.377 (0.232–0.612)	< 0.001*	0.949 (0.432–2.087)	0.896
TSAT (%)				
≤ 27.0	Reference			
> 27.0	0.593 (0.372–0.944)	0.028*		

*OR* odds ratio, *CI* confidence interval, *BMI* body mass index, *TIBC* total iron binding capacity, *TSAT* transferrin saturation.

### Development and validation of a novel logistic regression model for sarcopenia

**Figure 1:**
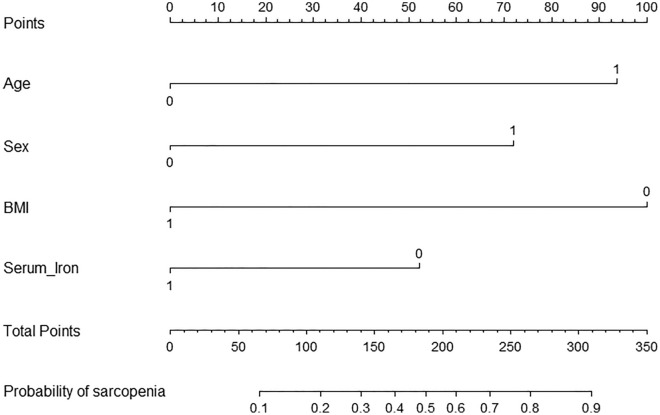
Nomogram of the logistic regression model for predicting sarcopenia. BMI = body mass index.

**Figure 2:**
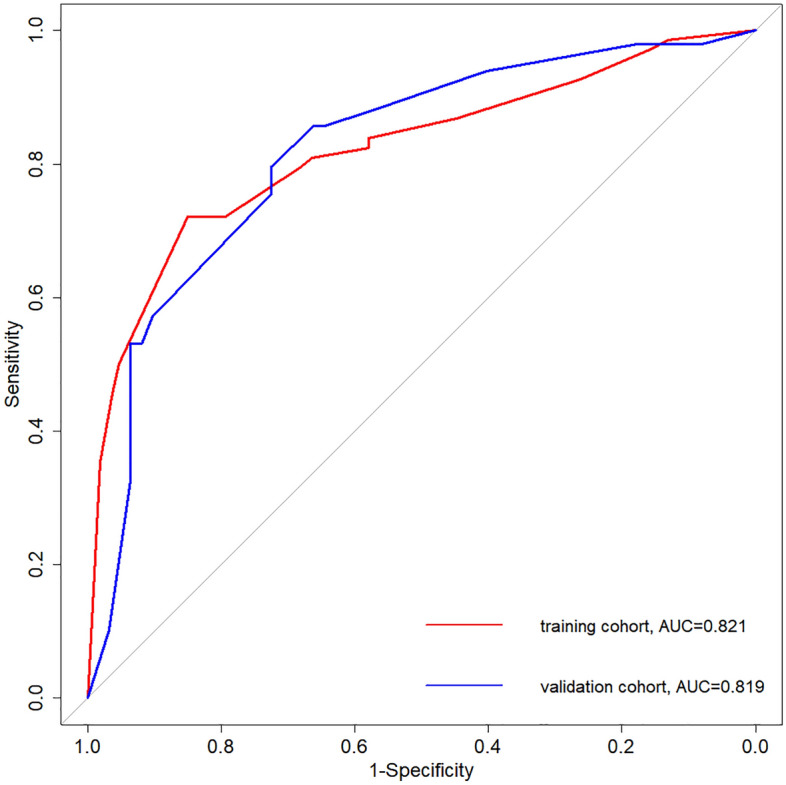
Calibration curves of the logistic regression model for prediction of sarcopenia. AUC = area under the curve.

The 286 patients were randomly assigned to a training cohort (n = 175) and a validation cohort (n = 111) in a 6:4 ratio, respectively. Sex, age, BMI, and serum iron level, which were previously confirmed to be significantly associated with sarcopenia, were included in the logistic regression model in the training cohort (Table 5). The nomogram of this model is shown in Fig. 1 and the calibration curves are presented in Fig. 2. The concordance index (C-index) for the new staging system was 0.821 in the training cohort and 0.819 in the validation cohort.

**Table 5:** Logistic regression model for prediction of sarcopenia in the training cohort.

Variable	Coefficient	OR (95% CI)	*P*
Sex, male vs female	1.296	3.65 (1.67–7.98)	0.001**
Age, > 65 years vs ≤ 65 years	1.687	5.4 (2.25–12.95)	< 0.001***
BMI, > 24 kg/m^2^ vs ≤ 24 kg/m^2^	− 1.799	0.17 (0.08–0.36)	< 0.001***
Serum iron, > 10.95 μmol/L vs ≤ 10.95 μmol/L	− 0.942	0.39 (0.16–0.93)	0.034*

*OR* odds ratio, *CI* confidence interval, *BMI* body mass index.

## Discussion

In this retrospective study, we found that (1) hospitalized patients with sarcopenia had decreased serum iron, transferrin, and TIBC levels compared to those without sarcopenia, and (2) serum iron remained an independent factor associated with the risk of sarcopenia in multivariate logistic regression analysis, after adjustment for relevant covariates. We developed and validated a logistic regression model for the prediction of sarcopenia, including sex, age, BMI, and serum iron level as independent variables.

Our results highlight the significance of iron status measurements in the prediction of sarcopenia, suggest iron metabolism as a contributor to sarcopenia pathogenesis, and indicate iron status as a possible target for treatment. It is generally accepted that a dysregulated iron status is involved in age-related muscle dysfunction or sarcopenia. Iron metabolism can affect muscle function by regulating mitochondrial function, ferroptosis, and insulin signaling, leading to decreased energy production and utilization^24–27^. Increased iron accumulation has been observed in the skeletal muscles of aged animals and has been suggested as a contributing factor to sarcopenia^20^. In aged rats, elevated levels of non-heme iron and related proteins, including ferritin, were found in skeletal muscles, indicating cellular iron overload (summarized in^28^). One human study directly investigated iron accumulation in skeletal muscle and arrived at similar conclusions^16^.

Despite the compelling link between dysregulated iron metabolism and skeletal muscle loss in animals, the association between iron status and sarcopenia in humans remains inconclusive. This is partly due to the lack of methods to directly study the iron overload in human skeletal muscle, as muscle biopsy cannot be performed on all occasions. Serum markers for iron metabolism provide an alternative, and serum ferritin levels have been widely used. Iron overload, as reflected by high serum ferritin levels, was reported as a risk factor for sarcopenia in several studies^13,15,16^. However, iron deficiency, reflected by low ferritin levels, was also reported to be associated with skeletal muscle abnormalities and inspiratory muscle weakness^11,12^. In iron-deficient myotubes, branch-chain amino acid and insulin-stimulated protein synthesis is attenuated, highlighting the importance of iron utilization for muscle function^17^. Iron intake was also found to be positively associated with physical performance in older adults^29^. Therefore, the correlation between serum ferritin and muscle function has been inconsistent in the current literature.

Although studies have suggested an association between ferritin levels and sarcopenia, no such association was observed in our study. In contrast, transferrin, TIBC, and serum iron levels were significantly lower in patients with sarcopenia. As serum ferritin is closely correlated with tissue iron stores, it is possible that patients with sarcopenia have an abnormal iron distribution rather than depleted iron stores. Transferrin, TIBC, and serum iron levels reflect the level of iron in circulation and extracellular transport, and its decrease may be compensated by the increase in iron accumulation in the skeletal muscle, resulting in a relatively stable total iron store, as reflected by serum ferritin levels. Therefore, in patients with sarcopenia demonstrating normal serum ferritin levels, there may still be dysregulation of iron metabolism, as suggested by the results of our study. Excessive iron is deposited in the skeletal muscle, resulting in skeletal muscle dysfunction and decreased iron in circulation (serum iron, transferrin, and TIBC).

In the present study, we developed and validated a novel predictive model for sarcopenia that included age, sex, BMI, and serum iron levels as independent factors. Serum markers for iron metabolism are easily accessible and less costly than DXA and may assist in the prediction of sarcopenia in patients unable to participate in walking, chair stand, and balance tests.

There are two major limitations of our study: first, the study population was a relatively small sample of Chinese hospitalized patients; second, this was a retrospective single-center study performed in a hospital in Southern China, which limits the diversity of our patients. Also, certain possible confounding factors including daily physical activity levels, and sleep duration could not be assessed in our study. Therefore, studies with larger sample sizes are required to confirm our findings. Moreover, the predictive ability of our logistic regression model must be verified in prospective longitudinal studies.

## Conclusion

Transferrin, TIBC, and serum iron levels decreased in hospitalized patients with sarcopenia, but serum ferritin levels did not. Age, sex, BMI, and serum iron level were independent risk factors for sarcopenia. Our study highlights the significance of dysregulated iron metabolism in the pathogenesis of sarcopenia and suggests that abnormal distribution rather than increased total iron stores may contribute to muscle dysfunction. Further studies are required to clarify these underlying mechanisms.

## Data Availability

The datasets used and analyzed during the current study are available from the corresponding authors upon reasonable request.

## Abbreviations

BMI: Body mass index
DXA: Dual-energy X-ray absorptiometry
ROC: Receiver operating characteristic
RSMI: Relative skeletal muscle index
TIBC: Total iron binding capacity
TSAT: Transferrin saturation
